# Development of pea protein-derived 3D foam scaffolds cross-linked with heat and tannic acid for cellular agriculture applications

**DOI:** 10.1016/j.crfs.2025.101155

**Published:** 2025-07-22

**Authors:** Woo-Ju Kim, Yoonbin Kim, Begum Koysuren, Nitin Nitin

**Affiliations:** a Department of Food Science and Biotechnology, Seoul National University of Science and Technology, Seoul, 01811, Republic of Korea; b Research Institute of Food and Biotechnology, Seoul National University of Science and Technology, Seoul, 01811, Republic of Korea; c Department of Food Science and Technology, University of California-Davis, Davis, CA, 95616, USA; d Department of Biological and Agricultural Engineering, University of California-Davis, Davis, CA, 95616, USA

**Keywords:** Cultivated meat, Scaffold, Alternative meat, Pea protein isolate, Tannic acid, Cross-linking

## Abstract

Plant protein-based, 3D-structured scaffolds that mimic the mechanical properties of conventional meat products while supporting cytocompatibility and cellular growth are essential for developing alternative meat products. This study evaluates the development of pea protein isolate (PPI)-derived 3D foam scaffolds for cellular agriculture using heat and tannic acid (TA) as cross-linking agents. Infrared (IR) spectroscopy and scanning electron microscopy (SEM) analyses demonstrated the effects of thermal and TA treatments on the cross-linking of the proteins and microstructure of the PPI foam scaffold. Textural profile analysis (TPA) revealed that the mechanical properties of the fabricated scaffolds are comparable to those of conventional meats and meat analogues, and the addition of TA increased the hardness and reduced the cohesion of the scaffold. The result of the *in vitro* digestibility test demonstrated that the scaffolds exhibited a free amino acid release profile similar to that of pea protein gels. The cytocompatibility of the 3D foam scaffolds was evaluated using C2C12 myoblast cells as a model cell line. Rapid adhesion and significant proliferation of the cells were observed on the 3D foam scaffolds. Overall, the findings of this study highlight the potential of plant-based, 3D foam scaffolds for cellular agriculture applications.

## Highlights

• 3D pea protein isolate (PPI) scaffolds were developed for cultivated meat.
• Heat and tannic acid were used as cross-linking agents to fabricate PPI scaffolds.
• The textural properties of the PPI scaffolds resembled those of conventional meat.
• The digestibility of PPI scaffolds was comparable to that of native plant proteins.
• The PPI scaffolds supported the attachment and proliferation of myoblast cells.

## Graphical abstract

**Figure:**
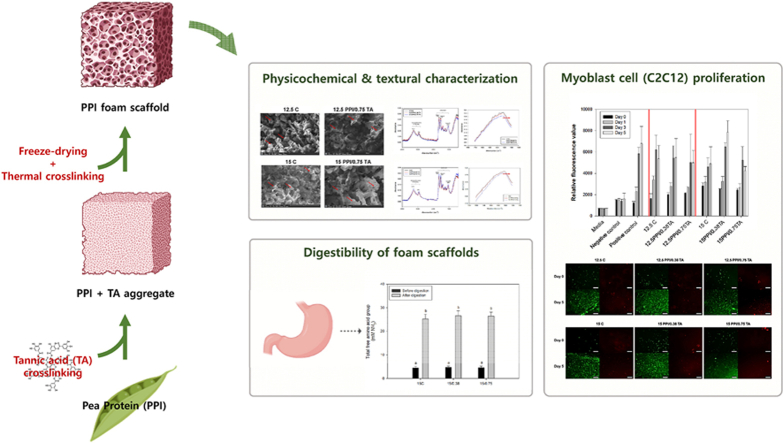

## 1 Introduction

Plant-based proteins are considered viable alternatives to conventional animal-based proteins (Maningat et al., 2022). Plant proteins can be obtained from various plant sources, such as legumes, seeds, and cereals, offering significant economic advantages (Kong et al., 2024). For example, it is estimated that the cost of producing 1 g of protein from plant sources, such as soybeans and wheat, can be ca. 4 to 32 times less expensive compared to those produced from animal sources, such as pigs and cows (Lusk and Norwood, 2009; Rubio et al., 2020). Therefore, extensive research has been undertaken to utilize plant proteins in producing plant-based meat alternatives, such as texturized vegetable proteins (TVPs) (Maningat et al., 2022). Beyond the applications in TVPs, plant proteins are gaining increasing interest as protein-rich, food-grade scaffolding materials for cultivated meat, offering opportunities to improve nutritional content, texture, and sustainability in alt-meat products (Ianovici et al., 2022; Wollschlaeger et al., 2022).

Scaffolds are 3D structures or frameworks designed to support the attachment, growth, and organization of mammalian cells (Levi et al., 2022). Scaffolds often mimic the extracellular matrix, such as collagen and laminin, found in the natural tissues, offering physical and biochemical cues to guide cell differentiation and tissue formation (Baker and Chen, 2012; Cai et al., 2020). Cytocompatibility, porosity, and mechanical strength are key properties of scaffolds for cultivated meat applications (Bomkamp et al., 2022; Kong et al., 2024). In addition, non-animal, food-grade materials that can provide a meat-like texture are desirable, as these scaffolds can be incorporated into the final product (Bodiou et al., 2020; Ren et al., 2008). Recently, several studies have demonstrated the potential of incorporating plant proteins into scaffolds for cultivated meat production (Ianovici et al., 2022; Wollschlaeger et al., 2022). In this approach, plant proteins are often blended with other biopolymers such as gelatin (Kovtun et al., 2015), alginate (David et al., 2024; Ianovici et al., 2022; Tansaz et al., 2018), chitosan (Ma et al., 2022), and pectin (Ianovici et al., 2022) in the form of hydrogels, microcarriers, or fibrous scaffolds (Bomkamp et al., 2022; Kong et al., 2024). However, due to the low mechanical strength of plant proteins, it is challenging to fabricate 3D structures solely composed of plant proteins or those with a high plant protein content (Y. Kong et al., 2023). For example, in our previous study, soy or pea proteins were incorporated into pectin-based gels (Kim et al., 2025). However, the protein content remained relatively low compared to the pectin fraction, as higher protein concentrations resulted in weaker textural properties. In addition, it has been reported that increasing the concentration of pea or soy protein above 1 % in polysaccharide-plant protein hydrogels results in decreased mechanical and rheological stability (Wollschlaeger et al., 2022). This constrains the amount of plant proteins that can be incorporated into the final product (<1 % in many cases), thereby limiting the nutritional and structural benefits that can be provided by the plant proteins (David et al., 2024). In addition, studies have demonstrated that the mechanical integrity of scaffolds can significantly enhance cell proliferation and differentiation, which are considered essential factors in developing cultured-meat products (Engler et al., 2004; Lapin et al., 2013; Ren et al., 2008). Therefore, the development of an effective food-grade strategy that enhances the structural integrity and mechanical stability of plant proteins in scaffolds is necessary to broaden their application in cultivated meat production.

Diverse physical and chemical cross-linking methods have been actively applied in the fields of biomedical and tissue engineering to stabilize protein structures (Alavarse et al., 2022; Indurkar et al., 2020). Among them, the use of synthetic cross-linkers, such as glutaraldehyde and carbodiimide, is considered the most effective and widely used method (Alavarse et al., 2022). However, only a limited number of methods are considered a food-grade approach that can be applied in the cultivated meat sector (Chng and Wan, 2023; Wei et al., 2023). Therefore, the use of natural cross-linkers such as polyphenols is attractive for cultivated meat applications. For example, phenolic compounds, such as tannic acid (TA), are capable of forming stable complexes with plant proteins mainly through hydrophobic interactions and hydrogen bonding (Li et al., 2020; Lomova et al., 2015). Furthermore, covalent cross-linking of protein molecules can be induced via protein-polyphenol conjugation under certain pH and temperature conditions (Balange and Benjakul, 2010; Quan et al., 2019). In addition to the chemical cross-linking methods, non-toxic, physical cross-linking methods, such as thermal processing, can further stabilize plant protein scaffolds by promoting protein denaturation and aggregation through diverse mechanisms, including alterations in hydrogen and disulfide bonds and/or electrostatic and hydrophobic interactions (Akharume et al., 2021; Suri et al., 2023; Xiang et al., 2022a, Xiang et al., 2022b; Xiang et al., 2022). However, to the best of our knowledge, the potential of utilizing a combination of food grade chemical and physical cross-linking methods to stabilize plant proteins and to formulate a 3D scaffold with high plant protein content has not been extensively explored (Gordon et al., 2025; D. H. Yang et al., 2025). Additionally, there is a knowledge gap in understanding the potential impact of polyphenol-thermal cross-linking on the mechanical strength, digestibility, and cytocompatibility of plant protein scaffolds.

Pea protein is one of the most widely used plant proteins in food applications due to its favorable amino acid composition, mild flavor, and functional textural properties (H. W. Choi et al., 2024; Schreuders et al., 2019). Pea protein provides a well-balanced amino acid profile, low allergenicity, and several health benefits, including antioxidant, antihypertensive, and anti-inflammatory effects (Burger et al., 2022; Z. X. Lu et al., 2020; Shanthakumar et al., 2022). In addition, its solubility and gelation properties are robust and comparable to those of soy protein (Z. X. Lu et al., 2020). Given its high water dispersibility characteristics, this study aimed to evaluate food-grade cross-linking strategies to develop stable 3D scaffolds and assess their ability to support the proliferation of a model myoblast cell line. To achieve this goal, 3D PPI foam scaffolds were formulated using the mold casting method. The effects of TA and heat treatment on the physicochemical and mechanical properties of the PPI foam scaffolds were characterized using Fourier transform infrared (FTIR) spectroscopy, scanning electron microscopy (SEM), and textural profile analysis (TPA). The functionality of the PPI foam scaffolds was evaluated based on their digestibility and cytocompatibility. The digestibility of the PPI foam scaffolds was evaluated upon *in vitro* gastric digestion. The cytocompatibility of PPI foam scaffolds in promoting the attachment and proliferation of mammalian cells was assessed using C2C12 cells (murine myoblast cells) as a model cell line. The findings of this study will provide valuable insights into developing plant protein scaffolds with high nutritional and textural properties, as well as desired cell attachment and proliferation.

## 2 Materials and methods

### 2.1 Materials

Pea protein isolate (PPI; 80 % protein, Terrasoul Superfoods, Fort Worth, TX, USA) was purchased at a local grocery market (Davis, CA, USA). Sodium chloride (≥99 %), glycerol (≥99 %), tannic acid (TA; ≥99.5 %), pepsin from porcine gastric mucosa (1064 units per mg protein), sodium tetraborate, sodium dodecyl sulfate (SDS), *β*-mercaptoethanol, and resazurin sodium salt were obtained from Sigma-Aldrich (St. Louis, MO, USA). Dulbecco's phosphate buffered saline (DPBS; pH 7.2) without calcium, magnesium, and phenol red was purchased from Thermo Fisher Scientific Inc. (Waltham, MA, USA). Hydrochloric acid (HCl) and ethanol were purchased from Fisher Scientific (Pittsburgh, PA, USA). Ultrapure water (18 MΩ cm) was obtained using the in-lab Milli-Q water ultra-purification system from EMD Millipore (Billerica, MA, USA).

### 2.2 Preparation of PPI foam scaffolds

**Fig. 1:**
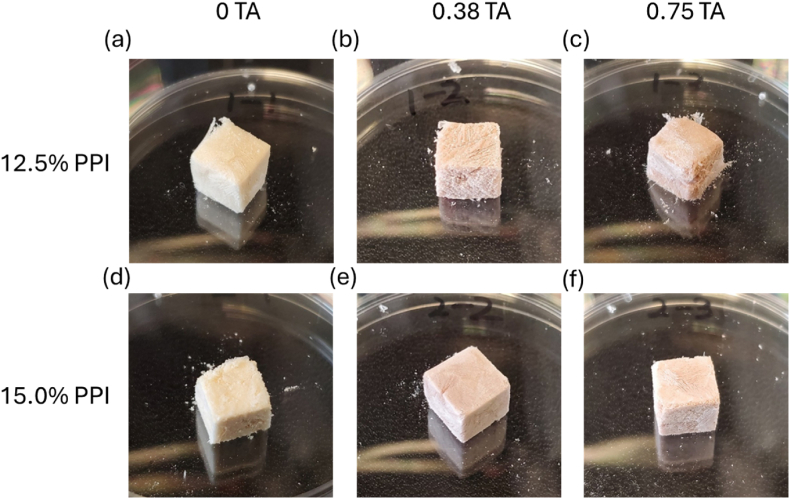
Images of pea protein isolate (PPI) foam scaffolds with and without tannic acids: (a) 12.5 C: 12.5 % PPI scaffolds with 0 % tannic acid, (b) 12.5PPI/0.38 TA: 12.5 % PPI scaffolds with 0.38 % tannic acid, (c)12.5PPI/0.75 TA: PPI scaffolds with 0.75 % tannic acid, (d) 15 C: 15 % PPI scaffolds with 0 % tannic acid, (e)15PPI/038 TA: 15 % PPI scaffolds with 0.38 % tannic acid, and (f) 15PPI/0.75 TA: PPI scaffolds with 0.75 % tannic acid. The proteins and tannic acid contents represent the initial concentrations used during gel preparation before freeze-drying and thermal cross-linking.

Different concentrations of PPI solutions (13.9 % and 16.7 %, w/w) with 5 % glycerol (w/w, based on PPI content) were prepared by dissolving PPI into 0.1 M NaCl solution. The solution was stirred for 1–1.5 h and stored in the refrigerator overnight to ensure sufficient hydration. Then, protein solutions of 12.5 % or 15 % were prepared with 0, 0.38 or 0.75 mg/mL TA. The concentration range was determined based on preliminary experiments (data not shown). To fabricate scaffolds with high protein content, protein dispersions with relatively high concentrations (12–15 %) were prepared after overnight hydration. Notably, a previous study reported that a minimum of 10 % pea protein suspension is required to form heat-induced hydrogels (Bartkuvienė et al., 2024). The mixtures of PPI solutions with different concentrations of TA were stirred for 1 min at room temperature, heated to 90 °C to denature the protein, and stored at 4 °C until further use. To create a foam scaffold, the protein solution was structured by using a customized mold as illustrated in Fig. S1. After freezing at −80 °C overnight, the scaffolds were freeze-dried to generate a porous structure. In this study, foam scaffolds refer to the macro-porous structures of PPI scaffolds formed through the freeze-drying process. Finally, the scaffolds were autoclaved to enhance the stability in water as illustrated in Fig. S2. The images of the foam scaffold are illustrated in Fig. 1. The abbreviations used in this study to describe PPI foam scaffolds with different compositions are summarized in Table 1.

**Table 1:** Abbreviations used for describing pea protein isolate (PPI) foam scaffolds prepared using 12.5 % or 15 % (w/w) PPI solution with different tannic acid (TA) concentrations.

Abbreviation	Description
12.5 C	12.5 % PPI solution with no TA
12.5 PPI/0.38 TA	12.5 % PPI solution with 0.38 mg/mL TA
12.5 PPI/0.75 TA	12.5 % PPI solution with 0.75 mg/mL TA
15 C	15 % PPI solution with no TA
15 PPI/0.38 TA	15 % PPI solution with 0.38 mg/mL TA
15 PPI/0.75 TA	15 % PPI solution with 0.75 mg/mL TA

### 2.3 Chemical and mechanical properties of PPI foam scaffolds

The chemical structural properties of the scaffolds were analyzed by using FTIR spectrometer. The spectra were in the range of 4000–400 cm^−1^. For the measurement, the spectrum was analyzed with 128 scans at a resolution of 4 cm^−1^ and a scan rate of 0.2 cms^−1^. The spectral data were subsequently baseline-corrected and smoothed, followed by second derivative processing to enhance peak detection. Identified peaks were then deconvoluted and curve-fitted using multi-Gaussian models. All data processing and spectral fitting were performed in Python 3.10 (Python Software Foundation, Wilmington, DE, USA) using the pandas, NumPy, SciPy, and matplotlib libraries. Protein secondary structure contents (%) were calculated based on characteristic band assignments in the amide I region (1600–1700 cm^−1^). Peaks at 1618–1630 cm^−1^ were assigned to β-sheet, 1645–1652 cm^−1^ to α-helix, 1640–1644 and 1655–1660 cm^−1^ to random coil, and 1670–1695 cm^−1^ to β-turn (Barth, 2007; J. Kong and Yu, 2007).

The total protein content of the foam scaffolds was measured based on the nitrogen content using the standard combustion method established by AOAC International (AOAC International, 2000). A conversion factor of 6.25 was applied to calculate the protein content from the nitrogen content with a detection limit of 0.1 % protein (dry basis). The microstructural properties of the cross-sectional area of foam scaffolds were also characterized by using scanning electron microscopy (SEM) (Thermo Fisher Quattro S). The scaffolds were sputter-coated with gold deposits under vacuum for 30 s, and the electron microscopy imaging was performed at 500× magnification, with a 5 kV accelerating voltage and a spot size of 3.0. To assess the textural properties of the foam scaffolds, cubic-shaped scaffolds (1 × 1 × 1 cm^3^, length × width × height) were prepared following the procedure described above (section 2.2). A texture analyzer (TA instrument, DE, USA) equipped with a TA-30A probe was used for the analysis. Each sample was placed at the center of the stage, and the measurements were performed at a speed of 1 mm/s with a strain of 50 % following the experimental protocol described by Chiang et al. (2019).

### 2.4 Digestibility of PPI foam scaffolds

The digestibility of the scaffolds was evaluated by measuring the free amino acid content after the *in vitro* gastric digestion of the scaffolds. Simulated gastric digestion was performed following the procedures described by Y. Lu et al. (2022) with slight modifications. Briefly, simulated gastric fluid (SGF) was prepared by adding 3.2 mg/mL of pepsin into 5 mg/mL of NaCl solution, and the pH of SGF was adjusted to 3.0 using HCl. A 0.1 g foam scaffold was placed in a 5 mL polypropylene tube and suspended in 2 mL SGF (pre-warmed to 37 °C). The tube was fixed horizontally in a rotary incubator and incubated at 37 °C with shaking at 100 rpm for 2 h. After incubation, the scaffold and SGF mixture was centrifuged at 13,000×*g* for 10 min, and the free amino acid content in the supernatant was extracted by mixing 100 μL of the supernatant (liquid digesta) with 1.5 mL of sodium tetraborate extraction buffer (0.0125 M with 2 % SDS; pH 9) (Mennah-Govela and Bornhorst, 2021), followed by bath-sonication (Branson 2510, 100 W, 42 kHz) at room temperature for 30 min.

The total free amino acid content in the extracted sample was measured using the *o*-phthaldialdehyde (OPA) method as described by Nielsen et al. (2001) and Ertugrul et al. (2021) with slight modifications. The free amino acid content released during digestion is a widely used indicator of digestibility and bioaccessibility of dietary proteins (Hall and Moraru, 2022; Jiménez-Munoz et al., 2023; Mennah-Govela and Bornhorst, 2021). Briefly, the OPA reagent was prepared by mixing 2 mL of OPA solution (40 mg/mL in 95 % EtOH), 50 mL of sodium tetraborate solution (100 mM; pH 9.3), and 200 μL of *β*-mercaptoethanol in a 100-mL volumetric flask, and the volume of the mixture was brought to 100 mL using deionized water (DW). Then, 1.0 mL of OPA reagent and 0.1 mL of sample solution were mixed, and the absorbance of the mixture was measured at 340 nm after 2 min of incubation at room temperature. A glycine solution (0.1–1.0 mg/mL) was used as a standard solution to obtain a calibration curve, and the free amino acid group in the liquid phase of the digesta was expressed in mmol NH_2_. The total amino acid content was estimated from the total protein content, assuming that the protein content approximates the total amino acid yield after complete hydrolysis. However, slight variations may occur due to differences in amino acid composition and nitrogen content (Boulos et al., 2020; Gorissen et al., 2018; Hayes, 2020). The degree of protein hydrolysis (DH, %) of the foam scaffolds was calculated using the following equation:

[1] $$DH\;\left(\%\right)=\frac{\left({NH}_{2\;After}{-NH}_{2\;Before}\right)}{Total\;{NH}_{2}}\times 100$$

where *NH*_*2 Before*_ and *NH*_*2 After*_ are the free amino acid content (mg) released into the liquid phase of the digesta before and after the gastric digestion of the scaffold (1 g), and *Total NH*_*2*_ is the total amino acid content (mg) of the foam scaffold (1 g).

### 2.5 Cell attachment and proliferation on PPI foam scaffolds

The cytocompatibility of the foam scaffolds was evaluated through cell attachment and proliferation assays. Mouse skeletal myoblasts (C2C12, ATCC CRL-1772) were used as the model cell line. Complete culture medium for C2C12 cells was prepared by supplementing basal medium (Dulbecco's Modified Eagle's Medium [DMEM]; high glucose, with pyruvate) with 10 % (v/v) fetal bovine serum (FBS) and 1 % (v/v) penicillin-streptomycin (PS; 100 × mixture).

For the cell attachment test, scaffolds were placed in a 48-well plate containing 500 μ l of DPBS and incubated at room temperature overnight to allow swelling. After incubation, the scaffolds were washed twice with DPBS. Cell suspensions containing 2 × 10^5^ cells (in 500 μL of complete media) were added to each well. After 1 min, the number of cells suspended in the complete media was enumerated by using a hemocytometer. The scaffolds were then incubated for an additional hour, and the number of cells remaining in suspension was enumerated. The attached cell population was calculated as follows:

[2] $$\text{Cell}\;\text{attachment}\;\left(\%\right)=\left(N_{0}-N_{t}\right)\;/\;N_{0}\times 100$$

where *N*_*0*_ is the initial cell population in media and *N*_*t*_ is the cell population remaining in media after incubation for the defined time (1 min or 1 h).

Proliferation of C2C12 cells on the foam scaffolds was assessed using a resazurin-based metabolic assay following the procedures described by Kim et al., 2024, Kim et al., 2025 with slight modifications. Briefly, C2C12 cell suspensions (2 × 10^5^ cells in 500 μl of complete medium) were added to the scaffolds and incubated at 37 °C with 5 % CO_2_ for up to 5 days. After incubation, 100 μl resazurin solution (0.15 mg/mL in DPBS) was added to each well and incubated at 37 °C with 5 % CO_2_ for an additional 1 h. Then, 100 μl of supernatant was collected, and fluorescence intensity was measured at 560 nm (excitation) and 590 nm (emission) using a SpectraMax M5 microplate reader (Molecular Devices, Sunnyvale, CA, USA). The viability of cells on the foam scaffolds was assessed using a Live/Dead cell imaging kit (Invitrogen, Carlsbad, CA, USA) according to the manufacturer's protocol. Briefly, scaffolds with proliferated cells were immersed in DPBS containing calcein AM and ethidium homodimer for 15 min and imaged using an inverted fluorescence microscope (Olympus IX-71, Olympus, Tokyo, Japan).

### 2.6 Statistical analysis

In the present study, statistical analysis was performed using JMP Pro 16 (JMP Statistical Discovery LLC, NC, USA). All experiments were performed at least in triplicates. The significant differences between treatments were determined through one-way or two-way analysis of variance (ANOVA) followed by Tukey's pairwise comparisons with a 95 % confidence interval (*p* < 0.05).

## 3 Results and discussion

### 3.1 Chemical and mechanical properties of PPI foam scaffolds

#### 3.1.1 FTIR spectroscopy

To fabricate the PPI foam scaffolds, PPI suspensions with or without tannic acid (TA) were heat-treated at 90 °C to induce thermal gelation. TA, a polyphenolic compound, can interact with pea proteins through hydrogen bonding and hydrophobic interactions, thereby promoting intermolecular cross-linking (Chen et al., 2022). In addition, TA can promote chemical crosslinking via reactions with functional groups, such as amines and thiols, through Michael addition or Schiff base formation (S. Kong et al., 2021). The resulting protein gels with or without TA were subsequently freeze-dried. During this process, sublimation of ice crystals generated an interconnected porous network, as characterized in the subsequent sections.

**Fig. 2:**
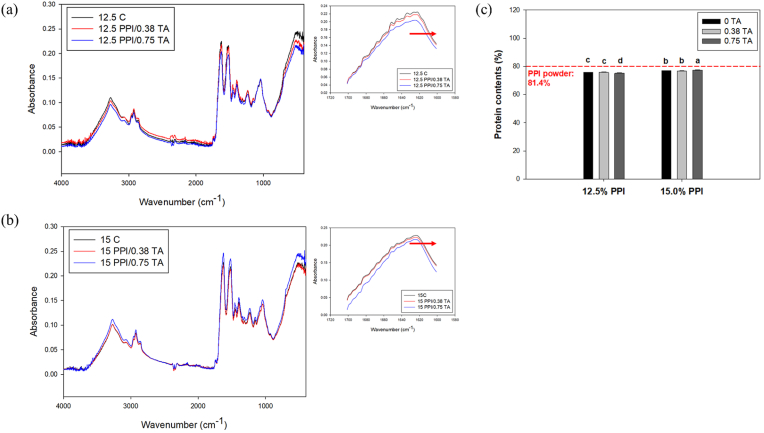
Fourier transform infrared (FTIR) spectra of (a) 12.5 % PPI and (b) 15 % PPI foam scaffolds with and without tannic acid, and (c) protein contents of the foam scaffolds. (a) 12.5 % PPI foam scaffold with and without tannic acid; (b) 15 PPI foam scaffold with and without tannic acid, and (c) protein contents of foam scaffolds. 12.5 C: 12.5 % PPI; 12.5PPI/0.38 TA: 12.5 % PPI +0.38 mg/mL of TA; 12.5PPI/0.75 TA: 12.5 % PPI +0.75 mg/mL of TA; 15 C: 15 % PPI; 15PPI/0.38 TA: 15 % PPI +0.38 mg/mL of TA; 15PPI/0.75 TA: 15 % PPI +0.75 mg/mL of TA.

Fig. 2a and **b** presents the FTIR spectra of PPI foam scaffolds. Characteristic peaks corresponding to amide I (1622 cm^−1^ NH bend stretch), amide II (1539 cm^−1^, NH bend stretch), and amide III (1234 cm^−1^, CN bend stretch) were observed, along with peaks associated with N-H vibrations (2956 cm^−1^) and O-H stretching (3273 cm^−1^). In previous studies, the presence of N-H vibrations and O-H stretching peaks has been linked to the formation of hydrogen bond networks within protein-based foam structures (Xiao et al., 2020). As the protein content of the foam scaffold ranges between 75 and 78 % (Fig. 2c), the amide I, II, and III peaks dominated the corresponding spectral regions at 1622 cm^−1^, 1539 cm^−1^, and 1234 cm^−1^, respectively (Nandiyanto et al., 2019). This finding is in agreement with the results of previous studies on plant protein scaffolds (Lawrie et al., 2007; Xiang et al., 2022). As shown in the spectra, the addition of TA resulted in only minor shifts in the spectral features, suggesting that FTIR spectroscopy may have limited sensitivity in detecting the cross-linking interactions between protein molecules induced by TA. This limitation may be particularly pronounced in this case, as denaturation of the protein at 90 °C could also induce significant cross-linking of the proteins. To further validate this, the heat-crosslinked and heat and TA-crosslinked scaffolds were submerged in water for an extended period. The non-crosslinked foam scaffolds dissolved rapidly before heating, while both the heat-treated and heat and TA-treated scaffolds retained their shape, as illustrated in Fig. S1. Similarly, in a previous study, heat treatment processing using an autoclaving process improved the mechanical properties of protein-based gel (Xiang et al., 2022). This may be due to the change in the ratio of protein secondary structures (alpha helix and beta sheet content) caused by thermal denaturation, resulting in a shift of the FTIR spectra, particularly in the range of 1700–1600 cm^−1^ (Lawrie et al., 2007; Xiang et al., 2022). Similar spectral shifts related to protein secondary structures were also observed from the heat-treated scaffolds, as illustrated in Fig. S3. Based on the spectral analysis of 12.5 % PPI scaffolds (Fig. S3d), slight increases in the β-sheet content and decreases in α-helix and random coil contents were observed from the heat-treated scaffolds. These secondary structural changes of pea proteins are consistent with previous reports (Lee et al., 2025; Liu et al., 2024; Moreno et al., 2020; S. Yang et al., 2025). For example, S. Yang et al., 2025, Yang et al., 2025 demonstrated that heat treatment of PPI at 100 °C resulted in a decreased α-helix content and an increased β-sheet content. The authors suggested that high temperature treatment may have led to the denaturation of the α-helix structures, which are then converted to β-sheet structures through intermolecular aggregation. However, no significant changes were observed among PPI scaffolds with different TA concentrations, indicating that TA had only a minor influence on the secondary structural changes of PPI scaffolds. The potential influence of TA on the morphology and texture of the scaffolds was further evaluated through SEM imaging and textural analysis in the subsequent sections.

#### 3.1.2 SEM imaging

**Fig. 3:**
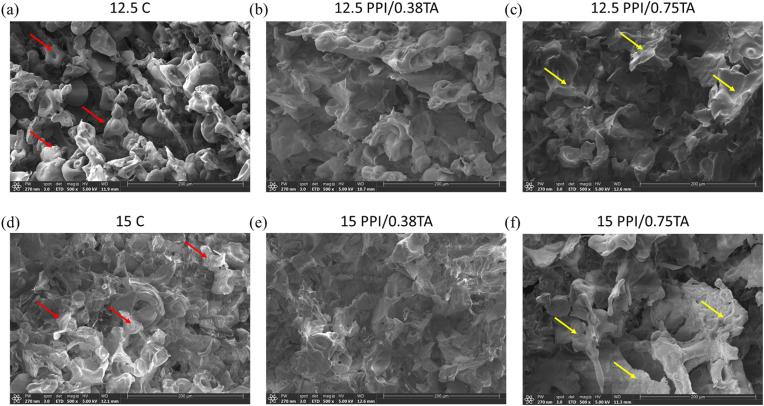
Scanning electron microscopy (SEM) images of pea protein isolate (PPI) foam scaffolds with and without tannic acid. (a) 12.5 % PPI (12.5 C); (b) 12.5 % PPI + 0.38 mg/mL of TA (12.5/0.38); (c) 12.5 % PPI + 0.75 mg/mL of TA (12.5/0.75); (d) 15 % PPI (15 C); (e) 15 % PPI + 0.38 mg/mL of TA (15/0.38); (f) 15 % PPI + 0.75 mg/mL of TA (15/0.75). Red arrow: dispersed and connected protein particles; Yellow arrow: clustered proteins. SEM images were taken at 500× magnification, and the scale bar indicates 200 μm.

Fig. 3 shows the microscale structural properties of foam scaffolds prepared with different concentrations of PPI (12.5 % and 15 %) and TA (0, 0.38, and 0.75 mg/mL). The images show the cross-sectional areas of the foam scaffolds. Overall, porous structures were observed within the foam scaffold after freeze-drying, regardless of the pea protein and TA concentrations. The presence and distribution of pores within the scaffolds are crucial parameters in the scaffold design. This is because pores can increase the surface area available for cell attachment and may facilitate the transport of nutrients to animal cells (Rao et al., 2023).

As illustrated in Fig. 3, TA content influenced the microstructure of the foam scaffolds. In the absence of TA (12.5 C and 15 C), the protein particles appeared as discrete particles that are linked together to form the scaffold structure. As described in section 2.2, the scaffolds were primarily formed through thermal denaturation of pea proteins following overnight hydration. During this process, the proteins did not fully dissolve but remained dispersed. Given the limited solubility of pea protein at high concentrations (12–15 %), heat treatment might have facilitated protein aggregation. Similar aggregation behavior has been reported for legume proteins in previous studies (Tiong et al., 2025). However, as TA content increases, particles tended to fuse together, resulting in a more continuous matrix with a defined pore architecture. Overall, these observations suggest that TA promotes the gelation of pea protein by acting as a cross-linker between protein molecules, thereby influencing both the microstructure and surface characteristics of the scaffold.

#### 3.1.3 Textural profile analysis (TPA)

As scaffolds can be consumed as a part of the cultivated meat products, it is desirable for them to exhibit textural properties comparable to those of conventional meat. In these hybrid food products, the mouthfeel can be significantly influenced by the scaffold (Seah et al., 2022). Hence, the textural properties of the foam scaffolds were evaluated. For the TPA, cubic-shaped (1 × 1 × 1 cm^3^, length × width × height) foam scaffolds were prepared.

Table 2 presents the results of the TPA performed on PPI foam scaffolds, including hardness, resilience, cohesion, springiness, gumminess, and chewiness at different PPI and TA concentrations. The results show that the addition of TA into PPI gels influenced the textural properties of the foam scaffolds. In particular, TA significantly influenced the hardness and cohesiveness of the foam scaffolds (*p* < 0.05). For example, hardness increased from 1.84 N to 3.84 N, as the TA content of 15 % foam scaffolds increased from 0 to 0.75 mg/mL. Conversely, cohesiveness decreased from 0.80 to 0.74 within the same range of scaffold composition. These effects may be attributed to the influence of TA on the microstructural properties of the foam scaffolds as illustrated in SEM images (Fig. 3). The hardness of these foam scaffolds was within the range reported for conventional meat products (1.56–16.09 N) (Mabrouki et al., 2023). However, cohesiveness was slightly higher than previously reported ranges for conventional meat products (0.35–0.77) (Ağar et al., 2016; Mabrouki et al., 2023).

**Table 2:** Texture profile analysis for scaffold foams after autoclave.

Scaffold gels	Hardness (N)	Resilience (%)	Cohesiveness	Springiness (%)	Gumminess	Chewiness
**12.5 C**	1.51 ± 0.14^ab^	41.1 ± 0.8^ab^	0.78 ± 0.01^ab^	55.6 ± 4.9^a^	1.15 ± 0.11^b^	0.64 ± 0.04^a^
**12.5/0.38**	2.18 ± 0.79^bc^	40.7 ± 2.8^b^	0.76 ± 0.04^abc^	54.0 ± 11.9^a^	1.62 ± 0.65^ab^	1.04 ± 0.47^a^
**12.5/0.75**	3.12 ± 0.81^c^	39.0 ± 2.9^ab^	0.71 ± 0.04^c^	51.4 ± 10.8^a^	2.24 ± 0.62^ab^	1.19 ± 0.58^a^
**15 C**	1.84 ± 0.57^bc^	44.7 ± 2.7^a^	0.80 ± 0.01^a^	56.3 ± 13.0^a^	1.71 ± 0.15^ab^	1.04 ± 0.16^a^
**15/0.38**	2.21 ± 0.42^bc^	39.8 ± 0.9^b^	0.76 ± 0.01^abc^	45.9 ± 8.7^a^	1.68 ± 0.33^ab^	0.68 ± 0.13^a^
**15/0.75**	3.84 ± 1.24^c^	41.6 ± 4.4^ab^	0.74 ± 0.04^bc^	52.3 ± 17.3^a^	2.74 ± 1.56^a^	1.24 ± 1.07^a^

a Means ± standard deviation from three replications. Values followed by the same letters within the column are not significant different (*p* > 0.05).

Contrary to hardness and cohesiveness, there was no evident trend or significant differences were observed in the resilience, springiness, gumminess, and chewiness of the foam scaffolds with the addition of TA (*p* > 0.05). However, the resilience (39–41.6 %) and springiness (45.9–55.6 %) of the foam scaffolds were within the reported range of commercial meat analogues and conventional meat products (Chang et al., 2011; Mabrouki et al., 2023). Similarly, the foam scaffold developed in the current study exhibited gumminess and chewiness comparable to those of conventional meat products. The gumminess (1.15–2.74) and chewiness (0.64–1.24) of the foam scaffold were within the range reported for conventional meat and meat analogues, which ranged between 0.52 and 9.18 and 0.49 and 7.42, respectively (Mabrouki et al., 2023). These observations highlight the potential of the developed foam scaffolds for use in cultivated meat products.

### 3.2 Digestibility of PPI foam scaffolds

**Fig. 4:**
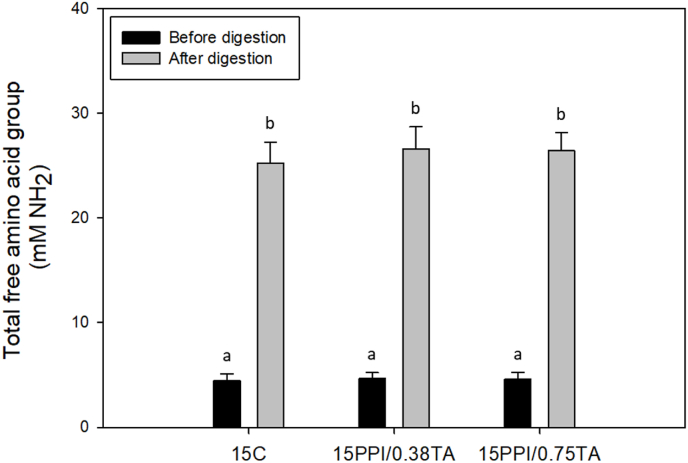
Digestibility of pea protein isolate (PPI) foam scaffolds during *in vitro* gastric digestion. PPI foam scaffolds were incubated in simulated gastric fluid for 2 h, and the total free amino acid content in the liquid digesta was measured before and after digestion. 15 C: 15 % PPI; 15/0.38: 15 % PPI +0.38 mg/mL of TA; 15/0.75: 15 % PPI +0.75 mg/mL of TA.

The digestibility of the foam scaffold was evaluated by measuring the free amino acid content released during the simulated gastric digestion (Fig. 4). Notably, a significant increase in free amino acid content was observed after 2 h of gastric digestion, and ca. 25.3–26.6 mM free amino acid groups were released into the liquid phase of the simulated gastric fluid. The degree of protein hydrolysis of 15 C, 15 PPI/0.38 TA, and 15 PPI/0.75 TA scaffolds were ca. 1.69 ± 0.18 %, 1.79 ± 0.22 %, and 1.76 ± 0.19 %, respectively. These results align well with those reported in previous studies on pea protein powders. For example, Santos-Hernández et al. (2020) reported that ca. 1.4 % and 2.4 % of free amino acids were released into the liquid digesta of garden pea and grass pea protein powders, respectively, after 2 h of gastric digestion. However, it is noteworthy that these values were obtained by digesting the PPI powders without any physical or chemical modification. This suggests that the TA and heat crosslinking used in the present study enabled the fabrication of 3D-structured pea protein scaffolds without compromising their digestibility. In addition, no significant differences (*p* > 0.05) in the digestibility were observed between scaffolds with varying TA concentrations. This may be attributed to the relatively low TA levels used in the present study (0.38 and 0.75 mg/mL). It is possible that these concentrations were not sufficient to significantly retard the proteolytic digestion of the scaffolds. This observation is further supported by secondary protein structure analysis (Fig. S3d), which showed no significant differences between the 12.5 % PPI scaffolds with varying TA concentrations. Similarly, a previous study conducted by Wang and Xiong (2021) reported that low TA concentrations did not significantly affect the digestibility of protein gels. Collectively, these findings suggest that the addition of TA enhanced the textural property (*i.e.*, hardness) of the foam scaffolds without negatively affecting their digestibility. The consistent digestibility of the foam scaffold can be attributed to its porous structure, which facilitates enzymatic interaction with proteases (Opazo-Navarrete et al., 2018). It is also possible that the thermal denaturation of pea proteins during heat treatment or autoclaving may have further improved their digestibility. However, in-depth analyses, such as INFOGEST or *in vivo* digestion studies, are needed to further understand the breakdown mechanisms of the foam scaffolds during digestion.

### 3.3 Cell attachment and proliferation on PPI foam scaffolds

**Fig. 5:**
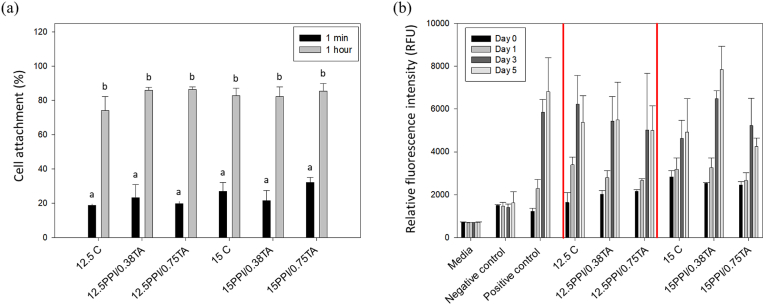
Illustration of animal cell (C2C12, mouse myoblast cell) (a) attachment and (b) proliferation for pea protein isolate (PPI) foam scaffold with or without tannic acid. 12.5 C: 12.5 % PPI; 12.5PPI/0.38 TA: 12.5 % PPI +0.38 mg/mL of TA; 12.5PPI/0.75 TA: 12.5 % PPI +0.75 mg/mL of TA; 15C: 15 % PPI; 15PPI/0.38 TA: 15 % PPI +0.38 mg/mL of TA; 15PPI/0.75 TA: 15 % PPI +0.75 mg/mL of TA.

**Fig. 6:**
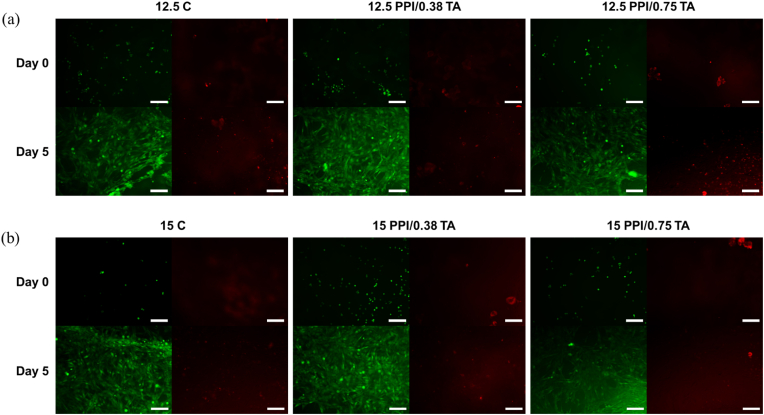
Illustration of Live/Dead staining for (a) 12.5 % pea protein isolate (PPI) foam scaffolds and (b) 15 % PPI scaffolds with animal cells during 5 days incubation. 12.5 C: 12.5 % PPI; 12.5PPI/0.38 TA: 12.5 % PPI +0.38 mg/mL of TA; 12.5PPI/0.75 TA: 12.5 % PPI +0.75 mg/mL of TA; 15C: 15 % PPI; 15PPI/0.38 TA: 15 % PPI +0.38 mg/mL of TA; 15PPI/0.75 TA: 15 % PPI +0.75 mg/mL of TA. Scale bars: 200 μ m.

Fig. 5 shows the attachment and proliferation of the C2C12 cells on PPI foam scaffolds. As illustrated in Fig. 5a, even a short period of incubation (1 min) promoted cell attachment, and ca. 18.8–32.0 % of cells were attached on the foam scaffolds. After 1 h of incubation, the number of cells attached to the surface of the foam scaffold significantly (*p* < 0.05) increased, and ca. 74.0–85.4 % of cells were attached on the scaffold surface. However, there were no significant (*p* > 0.05) differences between the foam scaffolds with different compositions. Following the cell attachment test, cell proliferation was evaluated over an extended incubation period (Fig. 5b). During 5 days of incubation, the foam scaffolds effectively supported the growth of C2C12 cells. For example, cells grown on 15 PPI/0.38 TA scaffolds showed strong fluorescence intensities comparable to those of the cells grown on a tissue culture plate (positive control) (*p* < 0.05). Further evaluation of the PPI foam scaffolds to support the cell proliferation of C2C12 cells was performed using microscopic imaging after Live/Dead staining of the cells (Fig. 6). At the initial stage of cell proliferation (Day 0), rounded and sparsely distributed C2C12 cells were observed. However, after 5 days of incubation, a confluent layer of myoblast cells was observed on the scaffold surface, exhibiting strong green fluorescence signals indicative of viable cells. Interestingly, the cells did not exhibit any particular orientation of growth on the scaffold surface, suggesting limited topographical or biochemical cues for cellular alignment within the porous structure. Despite the significant proliferation of viable cells, small specks of dead cells were also observed in the red fluorescence channel. This indicates the need for further studies to identify potential factors that can affect the cytocompatibility of the scaffolds, such as the generation of cytotoxic compounds during heat treatment or the presence of residual toxic compounds from the protein extraction process (Kim et al., 2025). Overall, these findings demonstrate good cytocompatibility of the scaffolds with C2C12 cells, supporting their potential application in muscle tissue engineering.

Scaffolds in tissue engineering have been fabricated using animal-derived materials such as collagen, gelatin, or their combinations, primarily due to their capacity to promote animal cell attachment and proliferation (D. J. Choi et al., 2018; Dong and Lv, 2016). This is largely attributed to the presence of specific amino acid sequences, notably the RGD motif, known to enhance cell adhesion, proliferation, and differentiation (Chimenti et al., 2011; Ruoslahti, 1996). Interestingly, a recent study has reported that plant proteins also contain similar sequences as the RGD sequences (Y. Kong et al., 2023). In particular, pea protein contains the bioactive tripeptide LRW, which can support the metabolic activity of animal cells, thereby facilitating their proliferation (Y. Kong et al., 2024). Although other materials present in plant materials might be less favorable for animal cells, these selective amino acid sequences can provide recognition sites for cell attachment and subsequent proliferation. The results from the present cytocompatibility test further support the potential of pea protein-based scaffolds to promote the growth of C2C12 myoblast cells. Future studies may be designed to investigate the interactions of these scaffolds with cell lines derived from different animal sources, including bovine, porcine, and aquatic species. Finally, the obtained results highlight the potential of fabricated 3D foam scaffolds for potential applications in cellular agriculture.

## 4 Conclusions

The study confirms the potential of a 3D foam scaffold composed of pea protein and tannic acid (TA) for cultivated meat production. TA-crosslinking, freeze-drying, and autoclave processes were involved in scaffold manufacturing. The chemical and mechanical properties of the scaffolds were analyzed based on FTIR, SEM, TPA, and simulated gastric digestion. The results demonstrated that the foam scaffolds were formed with porous structures that are essential for facilitating cell growth and nutritional delivery. Also, the 3D foam scaffolds exhibited textural properties and digestibility comparable to those of conventional meat proteins and plant proteins, respectively. Specifically, the hardness of the 15 % foam scaffolds ranged from 1.84 N to 3.84 N, and the degree of protein hydrolysis ranged from 1.69 % to 1.79 %. Finally, the 3D foam scaffolds effectively supported the adhesion and subsequent proliferation of C2C12 myoblast cells, showing no significant differences compared to the cells grown on a standard tissue culture plate (*p* > 0.05). These findings underscore the potential of pea protein–TA-based 3D foam scaffolds as cytocompatible, mechanically robust platforms for application in cellular agriculture, contributing to advancements in the cultivated meat industry.

## CRediT authorship contribution statement

**Woo-Ju Kim:** Conceptualization, Methodology, Validation, Formal analysis, Investigation, Writing – original draft, Writing – review & editing, Visualization, Project administration. **Yoonbin Kim:** Methodology, Validation, Formal analysis, Investigation, Writing – original draft, Writing – review & editing, Visualization. **Begum Koysuren:** Methodology, Formal analysis, Investigation. **Nitin Nitin:** Conceptualization, Methodology, Resources, Supervision, Writing – review & editing, Funding acquisition, Project administration.

## Declaration of competing interest

No conflict of Interest was declared.

## Data Availability

Data will be made available on request.
