# Supplementary material for: Development of pea protein-derived 3D foam scaffolds cross-linked with heat and tannic acid for cellular agriculture applications
Source: Curr Res Food Sci. 2025 Jul 22;11:101155. doi: 10.1016/j.crfs.2025.101155 (PMC12314388; doi:10.1016/j.crfs.2025.101155)

**Supplementary materials**

**Development of pea protein-derived 3D foam scaffolds cross-linked with heat and tannic acid for cellular agriculture applications**

Woo-Ju Kim ^a,b,c,1^_,_ Yoonbin Kim^c,1^_,_ Begum Koysuren^c,d^, and Nitin Nitin^c,d^*

^a^ *Department of Food Science and Biotechnology, Seoul National University of Science and Technology, Seoul, 01811, Republic of Korea*

^b^ *Research Institute of Food and Biotechnology, Seoul National University of Science and Technology, Seoul, 01811, Republic of Korea*

^c^ *Department of Food Science and Technology, University of California-Davis, Davis, California 95616, USA*

^d^ *Department of Biological and Agricultural Engineering, University of California-Davis, Davis, CA 95616, USA*

**Running title**: 3D scaffold based on pea protein for cultivated meat products

^1^ Co-first Authors

* Correspondence: Nitin Nitin, Department of Food Science and Technology, University of California-Davis, Davis, California 95616, USA. E-mail: nnitin@ucdavis.edu

**Figure S1.** Schematic diagram of customized mold used for structuring the protein scaffolds. The mold was fabricated using fused deposition modeling (FDM) with polylactic acid (PLA). The diameter of each mold was 6 mm, and the thickness of the mold was 4 mm.

**
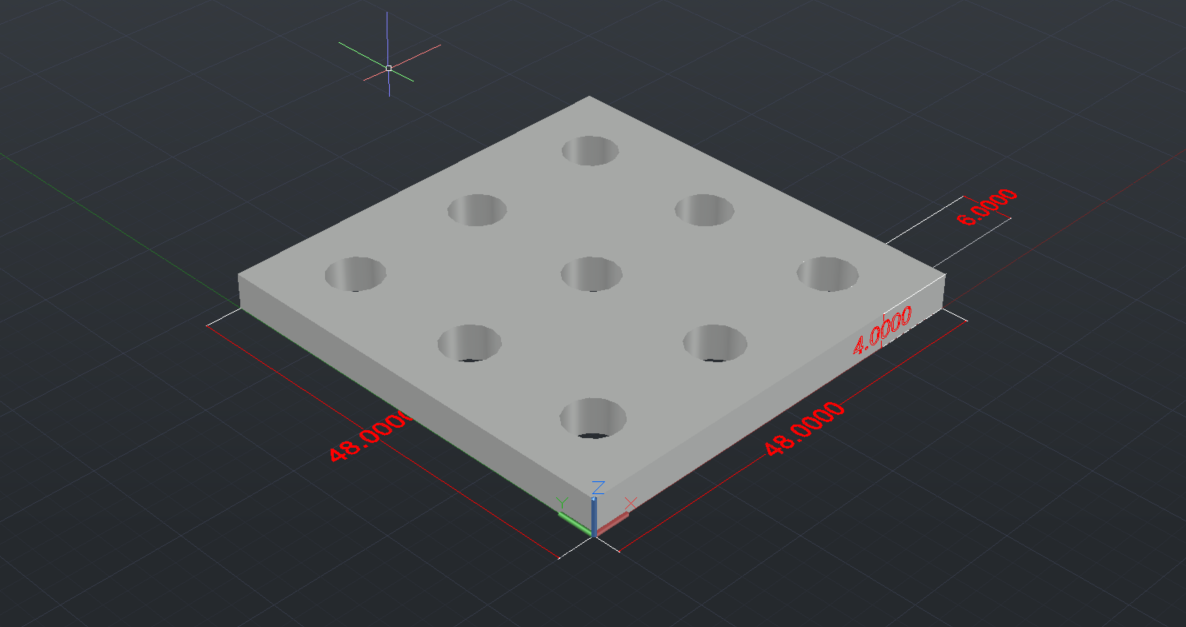
**

**Figure S2.** Pea protein scaffolds suspended in deionized water before (upper row) and after (lower row) autoclaving. 12.5 C: 12.5% pea protein isolate (PPI); 12.5/0.38: 12.5% PPI + 0.38 mg/mL of TA; 12.5/0.75: 12.5% PPI + 0.75 mg/mL of TA; 15C: 15% PPI; 15/0.38: 15% PPI + 0.38 mg/mL of TA; 15/0.75: 15% PPI + 0.75 mg/mL of TA.

**
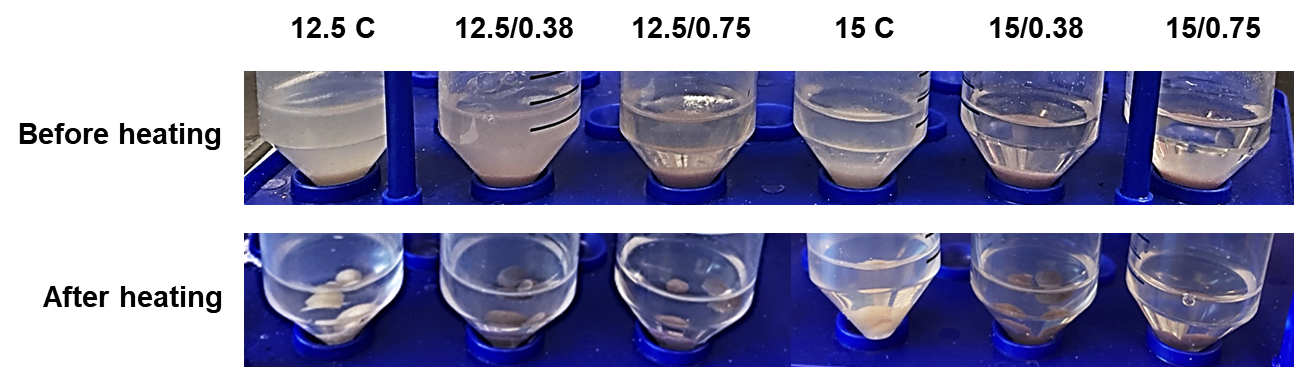
**

**Figure S3.** Fourier transform infrared (FTIR) spectra of 12.5% pea protein scaffolds with (a) 0% tannic acid, (b) 0.38% tannic acid, and (c) 0.75 tannic acid. 12.5 C: 12.5% pea protein isolate; 12.5/0.38: 12.5% PPI + 0.38 mg/mL of TA; 12.5/0.75: 12.5% PPI + 0.75 mg/mL of TA. (d) Secondary protein structures of 12.5% PPI scaffolds with (-H) and without (-NH) heat treatment


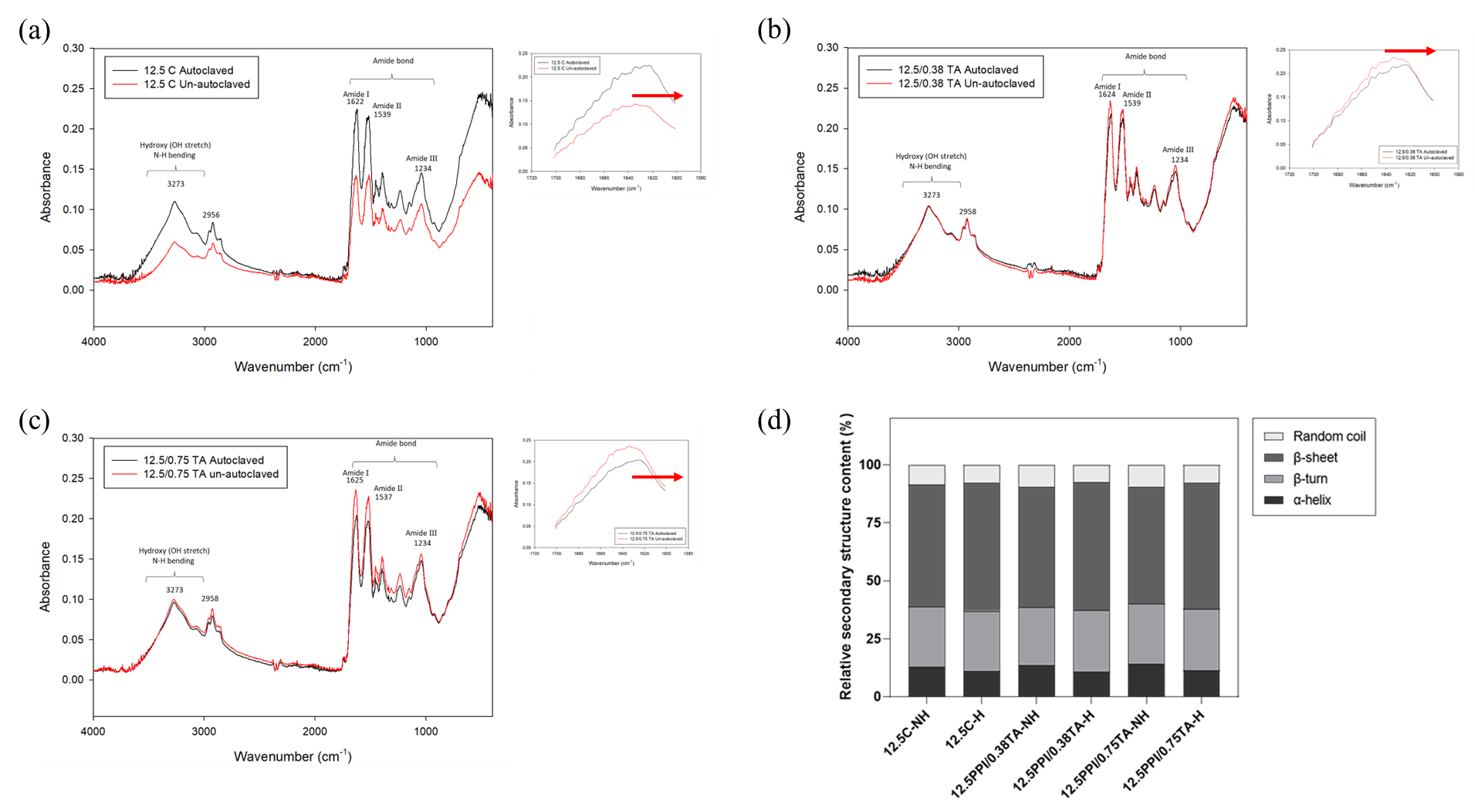

Supplement: Multimedia component 1 [file mmc1.docx]
